# RNA Polymerase III Regulates HIV Replication and Latency

**DOI:** 10.3390/v17091278

**Published:** 2025-09-20

**Authors:** Landon Thompson, Imran Jamal, Juthika Das, Casey Dang, Zhenzi Hong, Doran Katz, Alberto Bosque, Vir B. Singh

**Affiliations:** 1 Albany College of Pharmacy and Health Sciences, Albany, NY 12208, USA; landon.thompson@uky.edu (L.T.); imj17@pitt.edu (I.J.); juthika.das@acphs.edu (J.D.); caseydvdang@gmail.com (C.D.); zhenzi.hong@acphs.edu (Z.H.);; 2 School of Medicine & Health Sciences, George Washington University, Washington, DC 20037, USA; abosque@email.gwu.edu

**Keywords:** HIV latency, RNA Pol III, HIV reactivation

## Abstract

The elimination of HIV latent reservoirs is an extremely challenging task due to the interplay of multiple mechanisms regulating latency. Thus, we need to identify novel strategies to target heterogeneous reservoirs uniformly. Recent reports have provided intriguing evidence for the novel antiviral function of RNA Polymerase III (RNAP III), which remains to be further explored. In this study, we evaluated the role of RNA Pol III in regulating HIV latency and replication. We first demonstrated that the pharmacological inhibition of RNAP III can lead to a strong reactivation of latency in cell lines representing both T and monocytic cellular reservoirs. Next, we investigated the involvement of RNA Pol III in regulating HIV-1 replication using HIV-1 pseudotyped (DuoFluo) virus and HIV-1-Bal in THP-1 and Sup-T1 cells. We show that the pharmacological inhibition of RNAP III significantly induced HIV transcription. These findings were further confirmed in physiologically relevant primary CD4 T cells, and a consistent increase in HIV transcription was observed up to 72 h. Collectively, our study suggests that inhibition of RNAP III can increase the rate of HIV transcription, while the total HIV DNA remains unchanged. Overall, our study identifies a previously unknown role of RNA Pol III in restricting HIV transcription and advocates that targeting RNAP III-driven mechanisms could be a novel strategy to reactivate HIV latent reservoirs.

## 1. Introduction

Despite more than three decades of combination antiretroviral therapy (cART), complete eradication of human immunodeficiency virus (HIV) remains a daunting task. While cART has been very effective in limiting new cycles of infection and keeping viral load below or barely at detectable levels with the partial restoration of immune functions, it fails to provide a definitive life-long cure [1]. Evidently, interruption of cART leads to the quick rebound of viral load within a few weeks [2]. These observations attest to HIV’s ability to persist as undetectable latent reservoirs in a variety of tissues that remain insensitive to antiretroviral therapy. Characteristically, latently infected cells harbor integrated HIV provirus that is unable to actively produce viral transcripts, proteins, and virions, but it retains the potential to do so upon appropriate stimulation [3]. Activated CD4^+^ T cells are the primary target of HIV infection, and they eventually undergo cell death and release virus particles to further initiate another cycle of infection. However, a small fraction of infected CD4^+^ T cells may survive and eventually contribute to the persistent long-lived latent reservoir. Resting CD4^+^ T cells may also be nonproductively infected and remain dormant for several years [4]. Several studies during the past decade have demonstrated that a dynamic pool of latent cells can be maintained life-long via clonal expansion that is predominantly propelled by antigen-driven proliferation [1,5]. Latently infected cells circulate in peripheral blood as well as reside in a variety of tissues including lymph nodes [6], gut-associated lymphoid tissue (GALT) [7], the central nervous system [8], genital tracts [9], and bone marrow [10]. Although CD4^+^ T cells serve as the largest reservoir, a few other cell types have also been recognized to contribute to it, including monocytes, macrophages [11,12,13,14], and follicular dendritic cells [1].

At the molecular level, several mechanisms have been proposed for the establishment and maintenance of the latent state. Unlike other viruses (e.g., herpesviruses), HIV does not encode proteins that can regulate latency; rather, it depends on various cellular components that may be acting at various steps during infection to force HIV to undergo a nonproductive/latent infection. At early replication steps, HIV integration sites may dictate the fate of HIV expression [1,15]. HIV frequently integrates within intronic regions of actively transcribed genes, where proviral transcription might be hindered by host gene transcription via promoter occlusion or convergent transcription. Additionally, integration within repetitive elements in nongenic regions may render the provirus towards a repressive state [16,17]. The HIV promoter (5’LTR) is approximately 640 bp long and is wrapped around two nucleosome structures, namely nuc-0 and nuc-1. Numerous reports have suggested multiple epigenetic mechanisms controlling HIV expression [18]. A variety of chromatin modifications marking the repressive conformation of HIV promoter have been reported [1,18,19].

The “shock and kill” strategy to eliminate HIV reservoirs involves the reactivation of latent provirus by LRAs (latency reversing agents) followed by the induction of viral cytopathic effects and/or host immune response leading to the elimination of reactivated cells. This strategy typically supplements continuous cART to prevent de novo infection cycles caused by proviral reactivation, thereby achieving a complete cure. This approach has led to the identification of several LRAs including inhibitors of HDAC (Histone deacetylase), HMTs (Histone methyl transferases), DNMTs (DNA methyl transferases), Bromodomain and Extra terminal (BET) domain proteins, agonist for Protein Kinase C (PKC), and disulfiram (anti-alcoholism drug). Despite their successes recorded in vitro, these LRAs failed to eradicate the latent reservoirs clinically [20,21,22,23]. These failures can be attributed to an inherent heterogeneity in viral reservoirs within a particular cell type and across different cellular reservoirs, due to the compounding effect of multiple mechanisms that drive viral latency, cellular longevity, and modulation of host innate immune responses [24,25,26].

Interestingly, recent reports have suggested that RNA Polymerase III (RNAP III) may drive diverse mechanisms to support essential cellular functions as well as participate in the regulation of gene expression and antiviral response. RNAP III is known to transcribe a wide range of noncoding RNAs including 5SrRNA, tRNA, some microRNAs, and RNAs derived from SINEs (Small Interspersed Nuclear Elements), such as *Alu* sequences ranging from 100 to 300 nucleotides in length [27,28,29]. This molecule plays an important role in regulating gene expression and multiple cellular functions, most likely in a cell type-specific manner [30,31,32,33,34]. The expression of RNAP III transcribed genes is presumed to be stringently regulated, as dysregulation of these genes is implicated in multiple diseases, including cancer and neurological disorders [35,36,37]. Recently, a wide array of DNA viruses has been reported to induce RNAP III-mediated SINE activation [38,39]. Although these reports have stirred interest in identifying and characterizing the RNAP III transcribed genes, the cellular function of the RNAP III transcriptome remains largely unknown. These findings prompted us to employ both pharmacologic (RNA Pol III inhibitor ML60218) and genetic (siRNA-mediated knockdown of RNA Pol III) approaches to delineate the function of RNAP III in HIV expression. In this study, we observed that ML60218 treatment can achieve the strong reactivation of HIV in latent cell lines (J89GFP and THP89GFP). This is highly relevant, as the HIV genome preferentially integrates near *Alu* repeats that are often transcribed by RNAP III. We further investigated the role of RNAP III in controlling HIV-1 replication in a variety of cell lines. We observed that the knockdown or inhibition of RNAP III significantly induced viral replication post-integration. Collectively, this is the first study to investigate the role of RNAP III in regulating HIV-1 transcription, demonstrating consistent results in different cell types and providing a future direction to target RNAP III-driven mechanisms for eliminating and/or limiting the latent reservoir.

## 2. Materials and Methods

### 2.1. Ethics Statement

Primary CD4^+^ T cells were isolated from blood samples collected at the University of Rochester Medical Center (URMC) as part of a pilot study conducted during 2018–2019. The Research Subject Review Board at URMC approved studies involving human samples. All the study participants were healthy adults, and blood samples were obtained after written informed consent, in accordance with the declaration of Helsinki.

### 2.2. Reagents and Cell Culture

The RNA Polymerase III inhibitor, ML60218, was purchased from Millipore SIGMA (cat# 557403, Atlanta, GA, USA), and its latency reversing potential was tested on latently infected cell lines at 25 µM, 50 µM, and 100 µM concentration. Additionally, recombinant human TNF-α (10 ng/mL, R&D Systems, catalog number 210-TA/CF) and Vorinostat (SAHA; 10 µM, Medchem Express, catalog number HY-10221, Monmouth Junction, NJ, USA) were used as a positive control for latency reversal. We used latently infected monocytic (THP89GFP) and T cell lines (J89GFP). These cell lines were originally engineered by Dr. David Levy’s lab [40] and were received as a gift. These cell lines have been extensively characterized by multiple labs [41,42,43]. These cells were grown in RPMI 1640 medium (Sigma, cat# R8758-1L, Atlanta, GA, USA) supplemented with 10% fetal bovine serum, 100 U/mL of penicillin, and 100 µg/mL of streptomycin and were cultivated at 37 °C in a 5% CO_2_ atmosphere. THP-1 and Sup-T1 cells were maintained in RPMI medium with 10% fetal bovine serum, 100 U/mL of penicillin, and 100 µg/mL of streptomycin. HEK293T cells were cultured in DMEM medium (Sigma-Aldrich, cat#D5796, Atlanta, GA, USA) with 10% fetal bovine serum, 100 U/mL of penicillin, and 100 µg/mL of streptomycin. Human peripheral blood mononuclear cells were purified from the blood of healthy donors using density gradient centrifugation using Histopaque (cat# 10771, SIGMA-Aldrich). Further, CD4^+^ T cells were isolated using a Naïve CD4^+^ T cell isolation kit (cat#130-094-131, Miltenyi Biotec) and cultured in complete RPMI medium in the presence of IL-2 (30 U/mL). CD4^+^ T cells were stimulated with Dynabeads (αCD3/αCD28 beads, cat#14-307-D, Invitrogen) at a bead-to-cell ratio of 1:1 for 3 days. Cells were spinoculated with DuoFluo HIV (at a concentration of 100 ng/mL) for 2 h at 1200× *g* at 37 °C. Cells were cultured for up to 72 h and supplemented with IL-2 (30 U/mL).

RNA Pol III inhibition was performed using the inhibitor, ML60218, dissolved in di-methyl sulfoxide (DMSO) to 25 mM stock concentration, whereas treatments were given to achieve a working concentration of 25 μM, 50 μM, and 100 μM in cell culture media. In DMSO control conditions, an equal volume of DMSO was added compared to the treatment group. For infection studies, cells were pretreated with the inhibitor for four hours prior to infection. RNA Pol III knockdown was performed using siRNA targeting RNAP III (siPolr3a, Dharmacon Cat # M-019741-01-0005, Lafayette, CO, USA). Scrambled siRNA (Santa Cruz, cat # SC37007, Santa Cruz, CA, USA) was used as a non-target control. Both the siRNAs were used at a concentration of 10 µM.

### 2.3. RNA Extraction and Quantitative Reverse Transcription-Polymerase Chain Reaction (qRT-PCR)

Total cellular RNA was isolated using the Direct-zol^TM^ RNA MicroPrep kit (Zymo Research Corp, cat#R2062, Irvine, CA, USA). The cDNA was synthesized using the Transcriptor universal cDNA master (Roche, cat#45-58931510001, Atlanta, GA, USA). Quantitative real-time PCR reactions were performed using the iQTM SYBR Green super mix (Bio-Rad, catalog number 1708882, Los Angeles, CA, USA). HIV initiation transcripts were quantified using Trans-Activation Response (TAR) region-specific primers (HIV Initiation: Fw, 5′-GTTAGACCAGATCTGAGCCT-3′ and Rev: 5′-GTGGGTTCCCTAGTTAGCCA-3′). Elongated products were quantified using HIV Elongation (Fw, 5′-TGGGAGCTCTCTGGCTAACT-3′ and Rev: 5′-TGCTAGAGATTTTCCACACTGA-3′; these primers amplify the 140 bp region immediate downstream to the TAR element) and HIV Tat specific primers (Fw, 5′-ACTCGACAGAGGAGAGCAAG-3′ and Rev, 5′-GAGATCTGACTGTTCTGATGA-3′), as described earlier [44]. The 18S rRNA transcripts were amplified using a set of two primers (Fw, 5′-GCTACCACATCCAAGGAAGG-3′ and Rev, 5′-ACCAGACTTGCCCTCCAAT-3′). cDNA was quantified and normalized to the transcript levels of GAPDH (Fw, 5′-GTTAGACCAGATCTGAGCCT-3′ AND Rev, 5′-GTGGGTTCCCTAGTTAGCCA-3′). Additionally, total HIV DNA was measured using HIV-specific and beta-globin (internal control) primers, as described in a previous report [45].

### 2.4. Preparation of Pseudotyped HIV DuoFluo Virus

The Pseudotyped HIV-1 was prepared by co-transfecting HEK293T cells with HIV-1 DuoFluo reporter construct and Vascular Stomatitis Virus-protein G (VSV-G) construct. HIV DuoFLuo (R7GEmC) reporter construct was obtained through the NIH HIV Reagent Program (Cat# ARP-12595), Division of AIDS, NIAID, and NIH, contributed by Dr. Vincenzo Calvanez and Dr. Eric Verdin. Cell supernatants were collected after 72 h and filtered using the 0.45µ filter. The filtered virus was aliquoted and stored at −80 °C until use. Virus concentration was quantified by performing p24 ELISA (Abcam, Cat# ab218268, Boston, MA, USA). In vitro cultured cells were infected with DuoFluo HIV at a concentration of 100 ng per ml. DuoFluo HIV expresses two distinct reporters depending on if the infection is productive or latent. In this system, GFP expression is driven by an HIV promoter (5′-LTR), while mCherry is driven by a constitutive EF-1α promoter that functions independent of HIV promoter activity. Thus, GFP expression can be utilized as a marker of HIV expression using flow cytometry, whereas mCherry reporter is used to indicate nonproductive or latent infection [46].

### 2.5. Cellular Death Assays

Latently infected cells following respective treatments were stained with Annexin V using an Apoptosis detection kit (BD Biosciences, catalog number 556547), according to the manufacturer’s instructions. At least ten thousand events were acquired on FACSVerse (BD Biosciences) flow cytometer, and analysis was performed using FlowJo software (ver. 10).

### 2.6. Statistical Analysis

Each column indicates mean value, with error bars indicating the standard deviation. Two group comparisons were conducted by *t*-test, and multiple group comparisons were performed using One-way ANOVA and Dunnett’s multiple comparisons test using GraphPad prism software v9.5. * indicates *p* < 0.05, ** indicates *p* < 0.01, and *** indicates *p* < 0.0013.

## 3. Results

### 3.1. RNAP III Inhibition Promotes Latency Reactivation

RNAP III is one of the three RNA Polymerases present in eukaryotic cells. Besides its role in transcribing noncoding RNAs such as tRNAs, 5SrRNA, and U6 RNA, the full potential of RNAP III transcriptome remains unexplored. Recent reports have suggested the potential involvement of RNAP III in antiviral response by activating the transcription of repetitive elements and inducing the interferon signaling that may potentially converge on the regulation of HIV transcription and replication [38,39].

**Figure 1:**
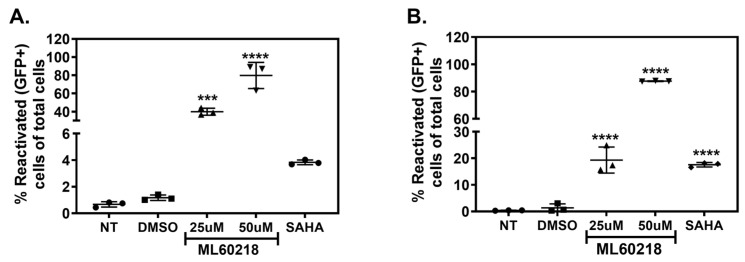
Inhibition of RNA Polymerase III causes latency reactivation. Levels of reactivated cells after 48 h of treatment with indicated doses of ML60218 were determined by measuring GFP+ cells using flow cytometry in THP89GFP (**A**) and J89GFP (**B**) cells. SAHA (10 µM) was used as a positive control. Each column shows the mean value, with error bars indicating the standard deviation *** Indicates *p* < 0.001, and **** indicates *p* > 0.0001. NT, non-treated; SAHA, Suberoylanilide Hydroxamic Acid. Circles, squares, and triangles are different shapes indicating number of biological replicates under the corresponding conditions.

**Figure A1:**
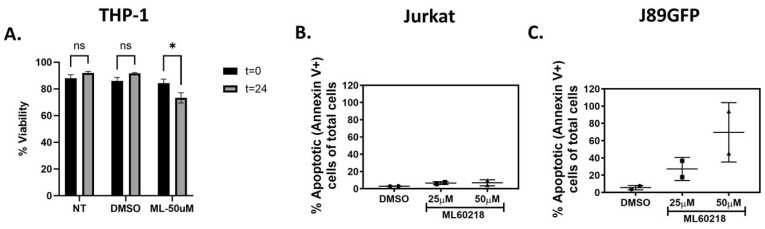
Effect of ML60218 on cell viability in uninfected THP-1 (**A**), Jurkat cells (**B**), and latently infected J89GFP cells (**C**). In THP-1 cells, the cell viability was measured by Trypan Blue Exclusion assay, while, in Jurkat and J89GFP cells, the death was measure by Annexin V assay. While minimal cell death was observed in THP-1 and Jurkat cells, the latently infected J89GFP cells showed massive cell death in response to ML60218-mediated reactivation. Each column shows the mean value with error bars indicating the standard deviation. ^ns^ indicates nonsignificant *p* > 0.05,* indicates *p* < 0.05.

**Figure A2:**
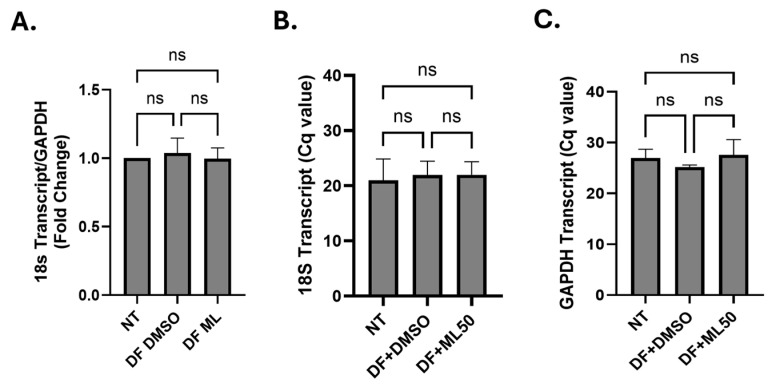
ML60218 specifically targets RNAP III without off-target effects on other RNA polymerases. (**A**) Levels of 18SrRNA were measured by RT-qPCR in primary CD4 T cells 24 h post-infection with DuoFluo HIV. GAPDH was used as the internal control. Further, 1 million live cells were collected from respective treatments (Nontreated, DMSO, and ML60218), and an equal volume of RNA was subjected to RT-qPCR using the primers specific to 18S rRNA and GAPDH transcripts. We plotted average Cq values from 3 independent experiments and included respective graphs in (**B**) (for 18S rRNA transcript) and (**C**) (for GAPDH transcript). The data suggest no significant effects of RNA Pol III inhibitor on 18S rRNA (Pol I transcribed) or GAPDH (Pol II transcribed). NT is nontreated (uninfected samples), DF is DuoFluo HIV, and ML50 is ML60218 (50 µM). Each column shows the mean value with error bars indicating the standard deviation. ^ns^ indicates nonsignificant *p* > 0.05.

To test this hypothesis, firstly, we investigated whether RNAP III inhibition can reactivate HIV in latently infected cell lines. We observed that the inhibition of RNAP III activity by ML60218 led to a strong reactivation of HIV in THP89GFP and J89GFP cells (Figure 1A,B, *n* = 3). Since RNAP III function is crucial for various essential cellular functions, it was imperative to test the effect of RNA Pol III inhibitor on cell viability. Thus, we investigated the effect of RNA Pol III inhibition on cellular apoptosis in uninfected THP-1 cells (Appendix Figure A1A, *n* = 3), Jurkat cells (Appendix A Figure A1B, *n* = 2), and latently infected J89GFP cells (Appendix Figure A1C, *n* = 2). Additionally, in order to evaluate off-target effects of ML60218 on RNA Pol I and RNA Pol II, we evaluated the effect of RNAP III inhibition on transcript levels of 18S rRNA (transcribed by RNA Pol I) and GAPDH (transcribed by RNA Pol II). Our results showed no significant changes on either of these transcripts (Appendix Figure A2), suggesting that ML60218 specifically inhibits RNAP III activity.

Further, RNAP III inhibitor does not cause any significant cell death in Jurkat and THP-1 cells, whereas the latently infected cells show heavy cell death consistent with the level of reactivation. These observations suggest that latently infected cells are more sensitive to ML60218. This could be explained by cell death as a result of HIV reactivation.

### 3.2. RNA Pol III Regulates HIV Transcription

**Figure 2:**
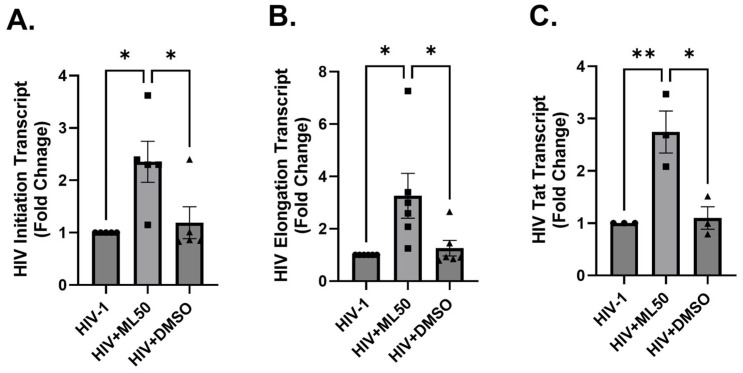
RNA Pol III inhibition induced the HIV-1 transcription in differentiated THP-1 cells. THP-1 cells were differentiated in the presence of 5 ng/mL PMA for 5 days. Differentiated cells were preincubated in 50 µM of ML60218 overnight and infected with HIV-Bal (equivalent to 2.33 × 10^8^ RT IU/mL) virus for 48 h. HIV transcription initiation (**A**), elongation (**B**), and Tat (**C**) specific transcripts were quantified by RT-PCR in cellular RNA. Each column shows the mean value, with error bars indicating the standard deviation. * indicates *p* < 0.05, ** indicates *p* < 0.01.

**Figure 3:**
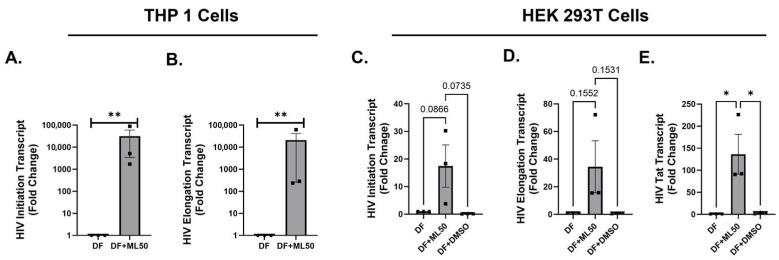
Inhibition of RNA Pol III causes an increase in expression of DuoFluo HIV transcription in THP-1 cells (**A**,**B**) and HEK293T cells (**C**–**E**). THP-1 cells were pretreated with 50 µM of ML60218 for overnight incubation and subsequently infected with DF HIV equivalent to 50 ng of p24 for 48 h. HIV transcription initiation (**A**) and elongation (**B**) specific transcripts were quantified by RT-PCR on cellular RNA. Further, HEK293T cells were similarly preincubated in 50 µM of ML60218 followed by infection with DF HIV. HIV transcription initiation (**C**), elongation (**D**), and Tat specific transcripts (**E**) were quantified in cellular RNA. Each column shows the mean value, with error bars indicating the standard deviation. * indicates *p* < 0.05, ** indicates *p* < 0.01.

After observing that RNAP III inhibition could reverse HIV latency, we decided to explore if blocking RNAP III could impact HIV replication in infected cells. Firstly, we tested differentiated THP-1 cells that attain a macrophage-like phenotype upon stimulation by phorbol 12-myristate-13-acetate (PMA). PMA differentiated THP-1 cells were infected with a wild-type HIV strain, HIV-Bal with or without 50 µM of the RNAP III inhibitor ML60218. DMSO was utilized in a third group as a loading control. Figure 2 shows that RNAP III inhibition by ML60128 significantly increased viral transcription. We showed that RNAP III inhibition resulted in increased levels of HIV initiation (Figure 2A), elongation (Figure 2B), and Tat transcripts (Figure 2C). Further, we tested the effect of RNAP III inhibition on HIV transcription in undifferentiated THP-1 cells. To achieve a significant infection, we utilized pseudotyped DuoFluo HIV to infect undifferentiated THP-1 cells. As seen in Figure 3A,B, the inhibition with ML60218 resulted in a considerable increase in the levels of HIV initiation and elongation specific transcripts. Similar results were obtained after the infection of HEK293T cells (Figure 3C–E) with VSV-G pseudotyped DuoFluo HIV, suggesting the consistent involvement of RNAP III-driven mechanisms in different cell types. Pseudotyping HIV with VSV-G envelope renders HIV infection of HEK 293T as it bypasses the need for HIV-specific receptors (CD4, CCR5, or CXCR4) and uses the wide tropism of VSV-G envelope [47,48].

**Figure A3:**
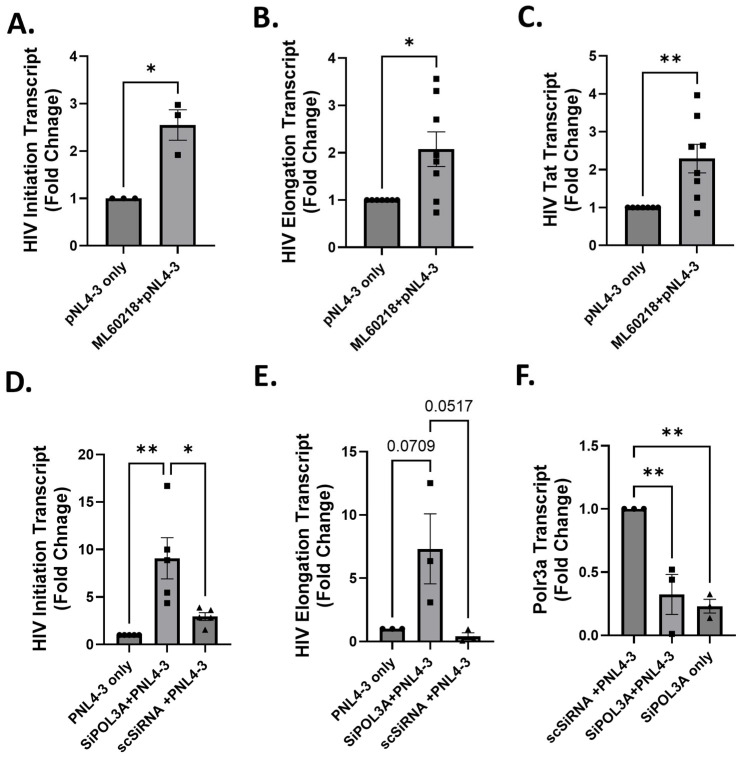
RNA Pol III inhibition (**A**–**C**) and knockdown (**D**–**F**) cause an increase in expression of pNL4-3 transfected in HEK293T cells. HEK293T cells were either preincubated with 50 µM of ML60218 overnight or transfected with 40 nM of siRNA specific to RNA Pol III. RT-PCR analyses on cellular RNAs show that the inhibition of RNA Pol III results in increased levels of HIV transcription initiation (**A**), elongation (**B**), and Tat (**C**) specific transcripts. Additionally, knockdown of RNA Pol III showed consistent results, showing an increase in the levels of HIV transcription initiation-specific (**D**) and elongation-specific (**E**) transcripts. We achieved the significant (more than 50%) knockdown of RNA Pol III using 40 nM siPOLR3a (**F**). scSiRNA, scrambled (non-target) siRNA. Each column shows the mean value with error bars indicating the standard deviation. * Indicates *p* < 0.05, ** indicates *p* < 0.01.

To specifically test the involvement of RNAP III in HIV transcription, we utilized the pNL4-3 ΔE EGFP construct. This plasmid contains the full-length HIV genome except for the envelope. We utilized HEK 293T cells to transfect the pNL4-3 construct, as HEK293T cells are routinely used to prepare large amounts of HIV-1 by transfecting them with a variety of HIV constructs, including pNL4-3, due to their high transfection efficiency and permissive cellular environment for HIV replication [49,50]. We used these cells to confirm the involvement of RNAP III in HIV replication by two complimentary manipulations of the RNAP III enzyme, i.e., pharmacological inhibition and siRNA-mediated genetic knockdown. Firstly, HEK293T cells were used to determine the effect of the pharmacological inhibition of RNAP. HEK293T cells were transfected with pNL4-3 ΔE EGFP plasmid, and viral transcripts were quantified 48 h post-transfection. Results showed a consistent increase in the viral transcripts in response to RNAP III inhibition (Appendix Figure A3A–C). This further supports the evidence that RNAP III may specifically regulate HIV transcription. Further, to address the concerns of possible off-site targets of ML60218 and to specifically attribute the increase in HIV transcription to the inactivity of RNAP III, we conducted gene knockdown assays using siRNA specific to RNAP III.

HEK-293T cells were transfected with siRNAP III (siPOL3A) or control siRNA for 48 h. Subsequently, cells were transfected with pNL4-3 HIV, and cells were harvested 48 h later to analyze the viral transcripts. Results demonstrated a significant increase in both initiation- and elongation-specific transcripts (Appendix Figure A3D,E) in response to approximately 50% knockdown in RNAP III transcript levels (Appendix Figure A3F).

### 3.3. RNA Pol III Regulates HIV Transcription in T Cells

**Figure 4:**
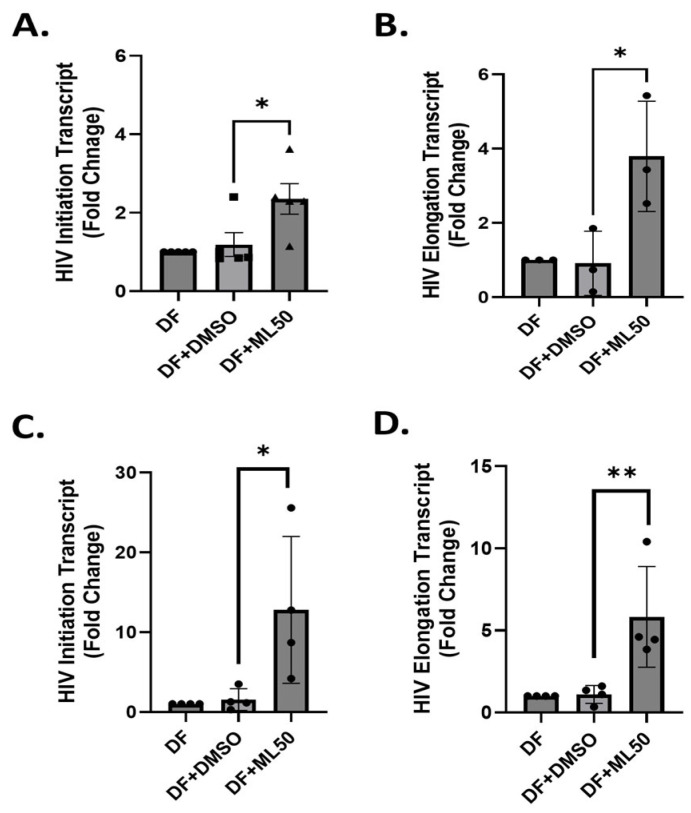
Pol3 inhibition promotes HIV-1 transcription in Sup-T1 Cells. SupT1 cells were infected with DuoFluo (DF) HIV only, pre-treated with DMSO (DMSO), or with ML60218 at 50 µM (ML50) for four hours. After four hours, pre-treatment cells were infected with DF virus for 6 h (**A**,**B**) and 24 h (**C**,**D**). The relative levels of cell-associated viral transcripts corresponding to HIV initiation and elongation are shown as fold change relative to DF using GAPDH as a housekeeping gene. * signifies *p* < 0.05 and ** signifies *p* < 0.01.

The fact that CD4^+^ T cells constitute the majority of latent reservoirs warranted replicating the above findings in a cell line representing T cells. For this, we utilized Sup-T1 cells that were infected with DuoFluo HIV (DF HIV) for 6 h and 24 h. Figure 4A,B shows the transcription status of DF HIV in Sup-T1 cells after 6 h of infection.

**Figure 5:**
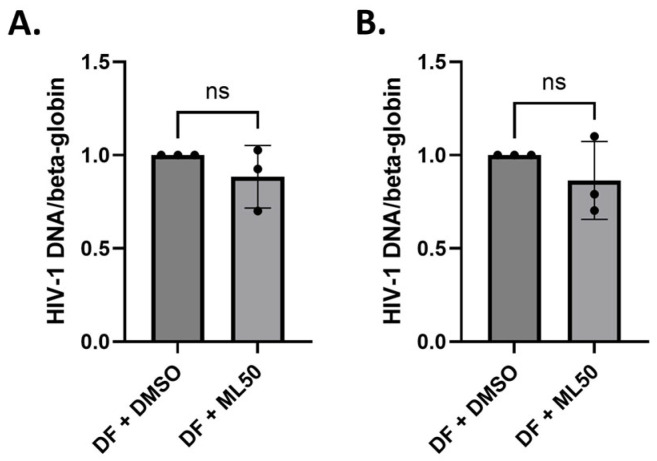
RNAP III inhibition does not alter total HIV DNA. HIV DNA was quantified 24 h post-infection by PCR using HIV-1-specific primers. Primers for beta-globin were used as an internal control to normalize the HIV DNA levels in Sup-T1 (**A**) and THP-1 (**B**) cells. DF, DuoFluo HIV; ML50, ML60218 at a 50 µM concentration. ns, not significant.

The results suggested that RNAP III inhibition caused a significant (2- to 5-fold) induction in HIV transcripts (initiation and elongation) within 6 h post-infection. The final timepoint analyzed for Sup-T1 cells was 24 h post-infection. Interestingly, we observed that HIV transcript levels remained elevated up to 24 h (Figure 4C,D). With the elevated levels of HIV detected, we speculated whether this increase is attributed to a higher infectivity rate (increase in the total HIV DNA) or specifically due to alteration in the transcription of integrated provirus. To address this, we further quantified the total amount of HIV DNA. In Figure 5, there are no statistically significant differences in the amount of total HIV DNA in Sup-T1 and THP-1 cells after 24 h. This suggests that RNAP III specifically modulates the expression of integrated provirus and does not affect the pre-integration steps.

**Figure 6:**
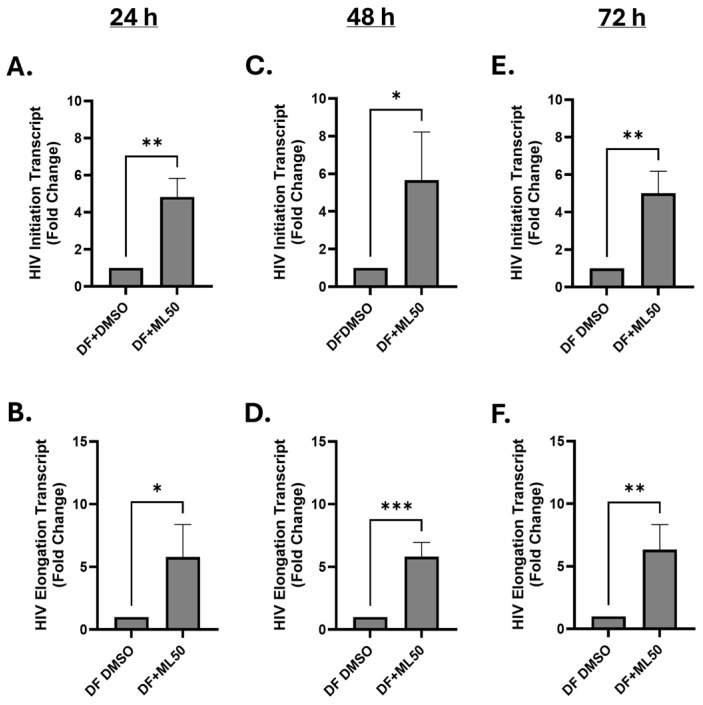
RNAP III inhibition promotes HIV-1 transcription in primary CD4^+^ T cells. Cells were infected with DuoFluo (DF) after pre-treatment with DMSO (DMSO) or with ML60218 at 50 µM (ML50) for four hours. Cells were cultured in the presence of 30 U/mL of IL-2 for up to 72 h. Cellular RNA was collected at 24 h (**A**,**B**), 48 h (**C**,**D**), and 72 h (**E**,**F**), and RT-qPCR was performed using HIV initiation- and elongation-specific transcripts (*n* = 5). Each column shows the mean value with error bars indicating the standard deviation. * Indicates *p* < 0.05, ** indicates *p* < 0.01 and *** indicates *p* < 0.001.

**Figure A4:**
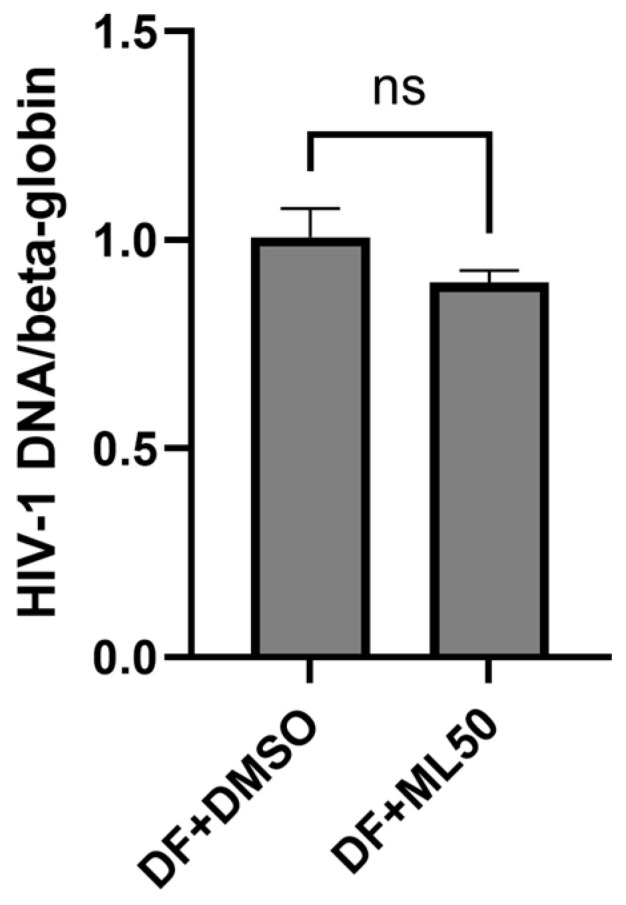
ML 60218 did not cause any significant changes in total HIV DNA. Total HIV-1 DNA was quantified by PCR at 24 h post-infection in primary CD4 T cells. Each column shows the mean value with error bars indicating the standard deviation. ^ns^ indicates nonsignificant *p* > 0.05.

**Figure A5:**
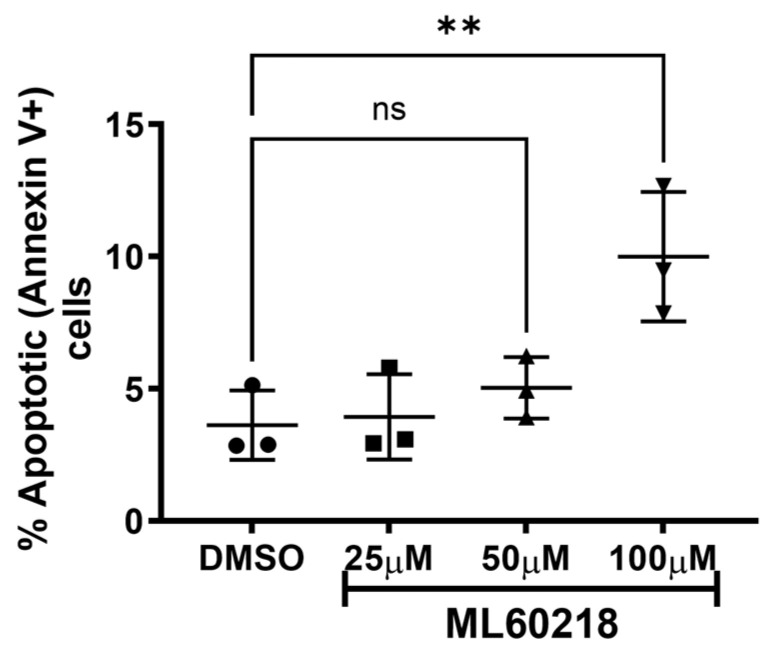
Level of apoptosis was measured by Annexin V assay in primary CD4 + T cells after 24 h of treatment with ML60218 at 25 µM, 50 µM, and 100 µM concentrations. Only the 100 µM concentration showed moderate cell death, whereas cells did not show any significant cell death up to the 50 µM concentration of ML. Each column shows the mean value with error bars indicating the standard deviation. ^ns^ indicates nonsignificant *p* > 0.05, ** indicates *p* < 0.005.

Further, we tested the effect of RNAP III inhibition on HIV replication in physiologically more relevant primary CD4^+^ T cells. We isolated CD4^+^ T cells from blood collected from three healthy donors and performed infection with DuoFluo HIV. We collected cells to measure HIV gene expression at three time-points, including 24 h, 48 h, and 72 h. Our results suggested a consistent increase in HIV transcript levels up to 72 h in response to pretreatment with ML60218 (Figure 6). Moreover, no differences in total HIV DNA were observed as measured at 24 h post-infection (Appendix Figure A4). Additionally, no significant cell deaths were observed up to 50 µM concentration of ML60218 (Appendix Figure A5). Altogether, our data suggest a potential role for RNAPIII in regulating HIV replication from early stages with a persistent long-term effect. Thus, in-depth understanding of RNAPIII driven mechanisms could bolster novel approaches for an HIV cure, specifically using the shock and kill strategy.

## 4. Discussion

RNAP III transcribes a variety of noncoding RNAs (5S rRNA, tRNAs, U6, Alu elements, 7SK, 7SL, and certain micro RNAs) that serve essential cellular functions, such as protein synthesis [27,28,29]. However, our study unravels the previously obscure function of RNAP III and identifies a novel mechanism to target the latent pool of HIV and develop a cure strategy. Pharmacological inhibition resulted in the induction of viral transcription, and this reactivation was coupled with cell death, potentially via cytopathic effects. This observation shows promise in devising a new approach to achieve a cure via the “shock and kill” strategy. However, antagonizing RNAP III is not a viable approach for “shock and kill” since RNAP III carries out essential cellular functions. Additional studies are warranted to tease out the complete signaling pathway and to identify intermediate molecules that can be targeted without having an adverse effect on essential cellular processes.

DNA viruses have been reported to induce the activation of SINE elements, specifically *Alu* sequences, which may impact host–virus interaction. These repeat sequences are generally transcribed by RNAP III [38,39,51]. Even though the exact mechanisms of the RNAP III-mediated transcription of *Alu* sequences are not fully understood, some reports suggest a crosstalk between RNAP II and RNAP III if located in genomic proximity [52,53]. In order to fully understand this scenario, we are conducting chromatin studies to identify RNAP III mediated changes at the HIV promoter in conjunction with RNAP III transcriptome and genomic occupancy in latent, reactivated, and productively infected cells. This approach will help us with the identification of novel effectors, including transcription factors and ncRNA with the potential to regulate cellular gene expression and restriction of HIV. Additionally, ML60218 has been well characterized as a novel broad-spectrum RNA Pol III inhibitor that is sensitive to changes in RNA Pol III conserved amino acid sequences, resulting in resistance to this inhibitor [54]. We have not been able to find any reports suggesting that this inhibitor can cause changes to the other two polymerases. However, depletion in tRNA and 5SrRNA levels may have an indirect effect on gene expressions due to the potential disruption of the protein synthesis process. A few reports demonstrated that Pol III inhibition or depletion may cause a shift in transcription at specific genomic loci, such as NANOG-Alu-Sx, which is typically transcribed by RNA Pol III, but the depletion or inhibition of RNA Pol III may allow the recruitment of RNA Pol II to this transcription unit [55,56]. Nevertheless, we conducted RT-qPCR analysis to quantify levels of 18S rRNA and GAPDH and found no significant changes in the expression of either of these transcripts that are transcribed by RNA Pol I and Pol II, respectively (Figure A2). Thus, we believe that ML60218-mediated changes to HIV expression are a direct consequence of RNAP III manipulation and are not caused due to an off-target effect.

Our acute infection studies in cell lines (THP-1 and SuP-T cells) and primary CD4 T cells indicated that the inhibition of RNAP III-mediated signaling may impact the establishment of latent reservoirs. Further, using HEK293T cells, we showed that the transcription rate of pNL4-3 plasmid was significantly increased by inhibition as well as the siRNA-mediated knockdown of RNAP III. Altogether, these data suggest that targeting RNAP III may enhance HIV transcription. Currently, there has been particular emphasis on using molecules that can promote viral replication during acute infection or early stages of cART, which may potentially limit the magnitude of latent reservoirs. Our findings in a variety of cell lines together suggest that inadequate RNAP III activity would promote productive infection that can potentially cause increased cell death in productively infected cells and not allow them to become quiescent, which eventually contributes to the establishment of a latent reservoir. However, further studies are needed to examine the effect of RNAP III on HIV during early events. Recently, RNAP III-transcribed ncRNAs have been shown to serve as pattern recognition receptor (PRR) ligands as they possess tri-phosphorylated 5′-ends [57]. Y-RNAs, one such class of RNAP III-transcribed RNAs, have been shown to specifically bind to RIG-I and induce the innate immune response against Measles Virus, Dengue Virus, and HIV-1. Specifically in the case of HIV-1, the detection of self-RNAs by innate sensors may cause the induction of interferon signaling, resulting in the restriction of HIV-1 replication and possibly the suppression of HIV, leading to latency [57]. Whereas, during chronic stages, this may explain sustained inflammation and immune exhaustion.

To add another dimension of complex pathways RNAP III might regulate, it has also been shown to transcribe viral genomic DNAs producing ncRNA that may promote viral replication and immune evasion [58,59]. These findings strongly advocate that RNAP III might regulate the expression of cellular genes as well as viral genes, and they can significantly impact host–virus interaction and have forced several viruses to evolve and encode strategies to manipulate RNAP III.

Our study utilized multiple immortalized cell lines to capture major aspects of HIV infection and latency. Although these cell lines represent multiple relevant cell types and offer considerable advantages with experimental manipulations, they do not fully reflect the physiology and heterogenous nature of latency in people living with HIV. To address this caveat, we intend to extend this study in a primary CD4^+^T cell model of HIV infection and latency. Further, key findings will be validated in cells isolated from aviremic patients on antiretroviral therapy. This approach will provide us with valuable insight into the behavior and response of the latent provirus to RNAP III manipulations. Additionally, our RNAP knockdown studies were conducted in HEK293T cells transfected with pNL4-3 construct, which may not fully represent the HIV infection model; thus, results need to be confirmed in a relevant infection model that represents either HIV latency or the transcription of an integrated provirus. Further, ML60218 may cause apoptosis at higher concentrations and longer exposure times that may induce HIV reactivation during early stages. We will address this possibility in our future studies. Nevertheless, our study presents compelling evidence and suggests a significant role for RNAP III-driven mechanisms in controlling HIV replication and latency that must be explored in depth to devise novel cure strategies.

## Data Availability

All the data concerning this study are included in this article. Additional information can be requested by sending an email to the corresponding author.
